# Case Report: Peritoneal dialysis-associated peritonitis caused by *Brucella*

**DOI:** 10.3389/fmed.2025.1722094

**Published:** 2025-12-04

**Authors:** Shu-fang Pan, Munire Abulizi, Adili Sawuti, Su-ying Han, Maimaitiaili Tuerxun, Jian-yun Zhu, Cai-lian Cheng

**Affiliations:** 1 Department of Infectious Diseases, The Third Affiliated Hospital of Sun Yat-sen University, Guangzhou, China; 2 Department of Infectious Diseases, The First People’s Hospital of Kashi Prefecture, Kashi, China; 3 Department of Nephrology, The Third Affiliated Hospital of Sun Yat-sen University, Guangzhou, China; 4 Department of Nephrology, The First People’s Hospital of Kashi Prefecture, Kashi, China

**Keywords:** brucellosis, *Brucella* peritonitis, peritoneal dialysis, peritonitis, BP

## Abstract

**Background:**

*Brucella* can affect multiple organs in the body, with peritonitis being a rare complication primarily observed in patients with cirrhosis or undergoing peritoneal dialysis. We aim to analyse the clinical features of patients with peritoneal dialysis-associated peritonitis to provide a reference for clinical diagnosis and treatment.

**Methods:**

A retrospective analysis was performed on three cases of *Brucella*-associated peritonitis in patients undergoing peritoneal dialysis, who were admitted to the First People’s Hospital of Kashi Prefecture between January 2022 and June 2025. The analysis covered general data, epidemiological history, clinical features, laboratory tests, and treatment efficacy.

**Results:**

All three patients had been in contact with animals or had drunk raw milk. Two were male and one was female. The patients were aged 36, 40, and 50 years old. They were all on peritoneal dialysis, and the main symptoms were abdominal pain, abdominal distension, and malaise, with no fever. All three patients had low leukocyte counts in their blood, a normal or mildly elevated neutrophil ratio, significantly elevated C-reactive protein levels, and mildly elevated procalcitonin levels. Peritoneal effluent showed >100 leukocytes/mm^3^ and a differential leukocyte count with a high proportion of mononuclear cells. *Brucella* was cultured from all peritoneal effluent samples, confirming the diagnosis of *Brucella* peritonitis. Symptoms decreased or disappeared following effective anti-infective treatment in all patients.

**Conclusion:**

*Brucella* peritonitis occurs in patients undergoing peritoneal dialysis and is characterised by abdominal pain and pressure. The basis for confirming the diagnosis is the presence of elevated leukocytes, predominantly monocytes, in the peritoneal effluent, and the culture of *Brucella* in the peritoneal effluent. Effective treatment involves a combination of doxycycline and rifampicin for at least 6 weeks, which controls peritonitis without requiring the removal of the peritoneal dialysis catheter.

## Background

Brucellosis is one of the most common zoonoses worldwide and is mainly transmitted through contact, the digestive tract, and the respiratory tract (1, 2). *Brucella* can affect multiple organs throughout the body, and peritonitis is a rare complication mainly found in patients with cirrhosis or undergoing peritoneal dialysis. It is usually characterised by abdominal distension, cloudy effluent, malaise, and nausea (3–5).

This paper retrospectively analyses three cases of *Brucella*-associated peritonitis in patients undergoing peritoneal dialysis who were admitted to the First People’s Hospital of Kashi Prefecture. The aim is to provide clinicians with a reference for managing similar diseases.

## Case present

### Case 1

A 40-year-old man was admitted to the hospital on 13 June 2022 with a 7-year history of proteinuria and 3 days of abdominal pain. He had undergone peritoneal dialysis on 18 February 2022 due to IgA nephropathy and stage 5 chronic kidney disease, and had started regular peritoneal dialysis treatment after the surgery, with regular follow-ups. Recent peritoneal dialysis regimen: 1.5% low-calcium peritoneal dialysis solution (2 L) three times a day, and 2.5% low-calcium peritoneal dialysis solution (2 L) left in the abdomen in the evening. The total ultrafiltration volume over 24 h is approximately 600 mL, and the daily urine volume is 200–300 mL. Three days ago, the patient developed abdominal pain and distension without any obvious triggers. There was paroxysmal distension of the whole abdomen, accompanied by fatigue, poor appetite, slightly turbid peritoneal dialysate, and smooth flow of peritoneal dialysate in and out. There has been no fever since the onset of the disease and no significant change in body weight. The patient enjoyed drinking raw sheep’s milk and had been doing so for half a month prior to the onset of the disease. The patient denied having any other chronic diseases and had no history of blood transfusion. Physical examination revealed that the superficial lymph nodes throughout the body were not palpably enlarged. The abdomen was soft with pressure and pain above the umbilicus; there was no rebound pain or muscle tension. There was no obvious redness, swelling, or secretion in the tunnel section or outlet of the peritoneal dialysis catheter.

His laboratory results show a white blood cell (WBC) count of 6.7 × 10^9/L, neutrophil percentage at 72.6%, lymphocyte percentage at 16.8%, hemoglobin (Hb) at 134 g/L, platelet (PLT) count at 172 × 10^9/L, alanine aminotransferase (ALT) at 33 IU/L, aspartate aminotransferase (AST) at 22 IU/L, albumin (ALB) at 36 g/L, urea nitrogen (Urea) at 16.73 mmol/L, creatinine (Cre) at 793 μmol/L, C-reactive Protein (CRP) at 60.52 mg/L, and procalcitonin (PCT) at 0.487 ng/mL. Dialysate routine analysis: colourless and slightly turbid; not coagulated; nucleated cells: 2170/mm^3^; mononuclear cells: 56.68%. Peritoneal effluent culture returned *Brucella*. Urological ultrasound showed diffuse changes in both kidneys. The prostate and bladder did not show obvious signs of obstruction or stones, and the ureter did not show obvious dilatation. Following the submission of the peritoneal effluent for routine analysis and culture, we commenced routine empirical treatment by adding 1 g each of cefazolin and cefotiam to 2 L of peritoneal effluent for intraperitoneal infusion. The peritoneal effluent culture yielded *Brucella*. In accordance with the advice of the Department of Infectious Diseases, the patient was prescribed rifampicin 0.45 g once daily orally and doxycycline tablets 0.1 g twice daily orally. He continued with his original dialysis regimen during the course of anti-infective therapy. His symptoms of abdominal pain and distension improved. The patient was discharged from the hospital and continued to take the above medication for 6 weeks. A peritoneal effluent culture taken in the outpatient clinic after 5 weeks was found to be free of bacterial growth. Twelve weeks later (6 weeks after stopping the medication), the peritoneal effluent routine was normal, and the peritoneal effluent culture was free of bacterial growth.

### Case 2

A 36-year-old man was admitted to the hospital on 26 June 2024 with a history of high creatinine levels for 2 years and abdominal pain for 1 day. He had undergone peritoneal dialysis catheterisation on 6 December 2023 due to stage 5 chronic kidney disease combined with underlying heart failure. He started peritoneal dialysis treatment after the operation and had regular follow-ups; he had no history of peritonitis. Once his renal function had recovered, it was recommended that the peritoneal dialysis catheter be removed. However, the patient refused because he was worried about his renal function deteriorating again. Consequently, he has been on low-dose peritoneal dialysis, using only two bags of fluid per day. Recent peritoneal dialysis programme: 1.5% low-calcium peritoneal dialysis solution (2 L) twice a day, flushing-based, with a daily urine output of 2000 mL. On 12 October 2023, he underwent surgery under general anaesthesia: total aortic arch replacement with artificial graft and elephant trunk vascular stenting (Sun’s procedure), aortic root replacement (Bental I), mitral valve repair, and temporary pacemaker placement. He was put on oral warfarin after the surgery, currently taking 5 mg per day with weekly dose adjustments according to the INR coagulation results. The patient developed abdominal pain 1 day ago, without any obvious triggers. He experienced paroxysmal, distending pain in the mid-abdomen, accompanied by fatigue, chills, a self-reported low-grade fever, clear transudate, and peritoneal dialysate flowing smoothly in and out of the peritoneal cavity. There has been no fever since the onset of the disease, nor any significant weight change. The suspected relevant epidemiological history was visiting the Sheep Bazaar (a live sheep trading market) and contact with cattle and sheep. On physical examination, superficial lymph nodes throughout the body were not palpably enlarged. With abdominal tenderness, mild tenderness on palpation around the umbilicus; there was no rebound pain or muscle tension. No obvious redness, swelling, or secretion was seen in the tunneled section of the peritoneal dialysis catheter or at the outlet.

His laboratory results include a WBC count of 5.09 × 10^9/L, neutrophils at 60.9%, lymphocytes at 20.4%, Hb at 125 g/L, platelets at 262 × 10^9/L, ALT at 16 IU/L, AST at 33 IU/L, Alb at 25.1 g/L, urea at 10.37 mmol/L, Cre at 306 μmol/L, CRP at 155.28 mg/L, and PCT at 0.337 ng/mL. Dialysate routine analysis: colourless, slightly cloudy, non-coagulable, contains 1,763 nucleated cells/mm^3^ and 55.5% mononuclear cells. The peritoneal effluent culture returned *Brucella*. No abnormalities were seen in the bilateral ureters, bladder, or prostate. In line with the advice of the Department of Infection, the patient was prescribed oral rifampicin 0.45 g once daily and oral doxycycline 0.1 g twice daily, which alleviated the symptoms of abdominal pain and distension. He was discharged from the hospital and continued to take these medications for 8 weeks. During the course of anti-infective therapy, he continued with his original peritoneal lavage regimen. After 2 months of follow-up at the clinic, his peritoneal effluent was found to be normal. The patient’s blood creatinine level was maintained at approximately 220 μmol/L, and his daily urine output was 2000 mL. Therefore, the abdominal cavity is currently flushed twice daily.

### Case 3

A 50-year-old woman was admitted to the hospital on 11 April 2025 with a history of fatigue and poor appetite for 3 years, and abdominal pain for 1 week. She had undergone peritoneal dialysis on 27 August 2024 due to stage 5 chronic kidney disease and had been undergoing regular peritoneal dialysis since the surgery. She had regular follow-ups and no history of peritonitis. Recent peritoneal dialysis regimen: 1.5% low-calcium peritoneal dialysis solution (2 L) four times a day, with a total ultrafiltration volume of approximately 800 mL over 24 h and a urine volume of 1,100 mL per day. One week ago, the patient developed persistent abdominal distension and pain, accompanied by malaise and chills. The peritoneal dialysis fluid was smooth going in and out, and the effluent was clear. There has been no fever since the onset of the disease and no significant weight change. The suspected relevant epidemiological history was that the patient’s husband sold raw meat and had contact with cattle and sheep. On 16 October 2024, she was diagnosed with brucellosis peritonitis (*Brucella* in peritoneal effluent culture) at our hospital. She was given oral rifampicin 0.6 g once daily for 8 weeks in combination with oral ceftriaxone 2 g once daily for 4 weeks for anti-infective purposes. The peritoneal effluent routine normalised on 5 November 2024 in our outpatient clinic. There was no history of other chronic diseases or blood transfusions. Physical examination: superficial lymph nodes throughout the body were not palpable or enlarged. There was abdominal softness and light pressure pain around the umbilicus, with no rebound pain or muscle tension. The peritoneal dialysis catheter tunnel section and outlet showed no obvious signs of redness, swelling, or discharge.

Patient laboratory tests reveal a WBC count of 7.14 × 10^9/L, neutrophils at 81.1%, lymphocytes at 13.0%, HGB level of 93 g/L, PLT count at 386 × 10^9/L, ALT at 17 U/L, AST at 3 U/L, Alb at 20 g/L, urea at 8.61 mmol/L, Cre at 444 μmol/L, CRP at 90.14 mg/L, and PCT at 0.273 IU/L. Dialysate routine analysis: colourless and slightly turbid; not coagulated; nucleated cells: 1611/mm^3^; mononuclear cells: 71.4%. Peritoneal effluent culture returned *Brucella*. Following the submission of the peritoneal effluent for routine analysis and culture, we commenced routine empirical treatment by adding 1 g each of cefazolin and cefotiam to 2 L of peritoneal effluent for intraperitoneal infusion. The peritoneal effluent culture yielded *Brucella*. In accordance with the advice of the Department of Infection, the patient was given moxifloxacin 0.4 g once daily orally and doxycycline 0.1 g twice daily orally, and her symptoms of abdominal pain and distension improved. The patient was discharged from the hospital and continued to take the above medications for 12 weeks. She continued with her dialysis regimen during the course of anti-infective therapy. Her peritoneal effluent was found to be normal after 2-month follow-up at the clinic. The patient is now free of abdominal pain and distension and continues to undergo regular peritoneal dialysis.

## Discussion

Kashi, in the Xinjiang region of north-west China, is a pastoral area with a high incidence of brucellosis (6). Brucellosis can affect several systems, including the osteoarticular, cardiovascular, and central nervous systems (7). Some patients can develop liver and spleen abscesses and peritonitis (8). *Brucella* peritonitis is rare and primarily occurs in patients with cirrhosis or renal failure undergoing peritoneal dialysis (3–5, 9).

We present three cases of patients undergoing peritoneal dialysis who did not have a fever. The main clinical manifestations were abdominal pain and pressure. By contrast, half of the four cases of cirrhosis-associated peritonitis that we previously summarised presented with a fever (10). This was considered to be related to underlying diseases such as cirrhosis or renal failure, which reduce the inflammatory response to infection. Therefore, we recommend performing blood cultures in patients with end-stage renal disease who are suspected of having brucellosis, regardless of whether they have a fever.

In these three patients, routine blood tests showed normal or mildly elevated neutrophil ratios, markedly elevated CRP, and mildly elevated PCT, with no evidence of high leukocyte counts. The peritoneal effluent showed more than 100 leukocytes/mm^3^ and a differential leukocyte count with a high proportion of mononuclear cells, which was similar in nature to tuberculous peritonitis. However, *Brucella* was cultured in the peritoneal effluent of all three patients, which confirmed the diagnosis of *Brucella* peritonitis. A positive *Brucella* culture is the gold standard for diagnosing brucellosis. The positivity rate of blood cultures fluctuates between 15 and 70%, and is potentially influenced by factors such as *Brucella* strain, disease stage, antibiotic use, and blood culture methodology (11, 12). *Brucella* bacteria grow slowly. The American Society for Microbiology and the World Health Organization (WHO) recommend a 4-week culture period involving blind subculturing of negative blood culture media. However, some studies suggest that conventional short-term cultures can detect *Brucella* within 3 to 7 days, eliminating the need for blind subculturing (12). Patients with a prolonged disease course and focal infections may require an extended culture period and terminal passage cultures to maximise *Brucella* isolation (12).

Common peritonitis associated with peritoneal dialysis is primarily caused by improper technique and exposure to contaminants, with Gram-positive cocci being the most prevalent (13, 14). Some scholars believe that *Brucella* may cause peritonitis by colonising catheters (15). However, *Brucella* has not been found to cause tunnelling or outlet infection of peritoneal dialysis catheters. It has also been established that patients with *Brucella* peritonitis can present with isolated peritoneal infections, i.e., negative serum agglutination tests and blood cultures, and positive serum agglutination tests and cultures of peritoneal dialysate. Therefore, cultures of peritoneal dialysate should also be performed in peritoneal dialysis patients with manifestations of peritonitis.

Chronic inflammation caused by *Brucella* infection is characterised by the infiltration of chronic inflammatory cells, such as lymphocytes and plasma cells (16, 17). This is in contrast to the neutrophil-dominant acute suppurative inflammation seen in other conditions. Consequently, peritoneal effusions during brucella peritonitis appear relatively clear, in contrast to the markedly turbid peritoneal effluent observed in suppurative peritonitis, which is characterised by massive neutrophil infiltration, bacterial presence, and necrotic tissue. Additionally, *Brucella* is an intracellular parasite that primarily survives and multiplies within immune cells, such as macrophages (18). The inflammatory response it triggers is relatively subtle, resulting in less direct damage to peritoneal tissue and less contamination of the effluent. Consequently, peritoneal effluent rarely exhibits a markedly cloudy appearance. So, if peritoneal effluent exhibits marked turbidity, caution should be exercised as there may be potential complications, such as concurrent infections (e.g., suppurative or tuberculous peritonitis) or progression of brucellosis, which can lead to tissue necrosis and increased exudation. Further investigation is necessary to determine the underlying cause.

Peritonitis caused by common bacteria is usually treated with intraperitoneal instillation over a period of 2 to 3 weeks (14, 19). For patients with brucella peritonitis, doxycycline combined with rifampicin is the recommended treatment, with quinolones or ceftriaxone as alternatives (20). Treatment with sulfonamides or aminoglycosides is not recommended in cases of renal failure. Conversely, rifampicin and doxycycline are primarily metabolised in the liver, so no dose adjustment is required in patients with renal insufficiency. According to the current literature, a combination of doxycycline and rifampicin for at least 6 weeks is an effective treatment for controlling peritonitis without the need to remove the peritoneal dialysis catheter in patients with a confirmed diagnosis of brucellosis peritonitis (4, 5, 21).

The patient in case 2 had undergone total aortic arch prosthetic vascular replacement and was at risk of infective endocarditis; the course was therefore extended to 8 weeks, despite negative blood cultures. Additionally, the patient in case 3, who experienced a second episode of brucella peritonitis, had an extended course of 12 weeks. For peritonitis caused by the same organism within 4 weeks, the likelihood of recurrence is considered high (22, 23). In this case, however, the patient had a 6-month interval between the two episodes and an epidemiological history of exposure to cattle and sheep, making reinfection a high possibility. Therefore, although peritoneal dialysis is a risk factor for peritonitis, patients who have recovered may still experience reinfection if the source of infection is not eliminated or the route of transmission is not interrupted.

The early symptoms of brucellosis are non-specific, often resulting in a delayed or incorrect diagnosis. Without timely treatment, the bacteria can multiply persistently within the body and invade multiple organs, causing a chronic infection. Whilst the overall mortality rate amongst brucellosis patients is low, the disease primarily manifests as long-term joint pain, fatigue, and neurological dysfunction, which can severely impact daily life and work capacity. A differential diagnosis of brucella peritonitis should be considered when patients undergoing peritoneal dialysis present with symptoms of peritonitis that do not respond well to initial anti-infective treatment, particularly in regions where brucellosis is endemic. Peritoneal dialysate culture, blood culture, and *Brucella* agglutination testing should be performed as early as possible. If necessary, the peritoneal dialysate should be examined using high-throughput sequencing technology to improve the accuracy of diagnosis. As the Kashi region of Xinjiang has a high tuberculosis prevalence, if the pathogen is not identified, tuberculosis peritonitis can be screened using a tuberculosis smear.

## Conclusion

*Brucella* peritonitis, which is characterised by abdominal pain and pressure, is seen in patients undergoing peritoneal dialysis. Confirmation of the diagnosis is based on the presence of elevated leukocytes, predominantly monocytes, in the peritoneal effluent, and the culture of *Brucella* in the peritoneal effluent. Positive blood cultures or test tube agglutination combined with manifestations of peritonitis also support a diagnosis of *Brucella* peritonitis. A combination of doxycycline and rifampicin for at least 6 weeks is an effective treatment for peritonitis, with no need for dosage adjustment in patients undergoing peritoneal dialysis.

## Data Availability

Publicly available datasets were analyzed in this study. This data can be found here: the original contributions presented in the study are included in the article. Further inquiries can be directed to the corresponding authors.

## References

[1] Khairullah A. R. Kurniawan S. C. Puspitasari Y. Aryaloka S. Silaen O. S. M. . Brucellosis: unveiling the complexities of a pervasive zoonotic disease and its global impacts. Open Vet J. (2024) (cited 2024 Aug 6);14. Available online at: https://pubmed.ncbi.nlm.nih.gov/38938422/10.5455/OVJ.2024.v14.i5.1 PMC11199761 38938422

[2] Narimisa N. Razavi S. Masjedian Jazi F. Risk factors associated with human brucellosis: A systematic review and Meta-analysis. Vector Borne Zoonot Dis. (2024) (cited 2024 Aug 6);24. Available online at: https://pubmed.ncbi.nlm.nih.gov/38597916/10.1089/vbz.2023.0092 38597916

[3] Al Zabali SM Rubaihan AK Alnetaifat MF Alshahrani S Alhammadi M. *Brucella* peritonitis in a patient on peritoneal dialysis: a case report and review of literature. Cureus. (2021) 13:e20679. doi: 10.7759/cureus.20679, PMID: 35106220 PMC8786575

[4] Al-Qarhi R. Al-Dabbagh M. *Brucella* shunt infection complicated by peritonitis: case report and review of the literature. Infect Dis Rep. (2021). (cited 2024 Aug 7). 13. doi: 10.3390/idr13020035 PMC8167664 33919608

[5] Huang Y. Zhu X. Shen W. Wang Y. Han M. Brucellosis-induced peritonitis and abdominal aortitis in a non-endemic area patient on peritoneal dialysis: a case report and literature review. Front Med. (2024) (cited 2024 Aug 6). 11. Available online at: https://www.ncbi.nlm.nih.gov/pmc/articles/PMC11160839/10.3389/fmed.2024.1393548 PMC11160839 38854664

[6] Chen X Emam M Zhang L Rifhat R Zhang L Zheng Y. Analysis of spatial characteristics and geographic weighted regression of tuberculosis prevalence in Kashgar, China. Prev Med Rep. (2023) 35:102362. doi: 10.1016/j.pmedr.2023.102362, PMID: 37584062 PMC10424202

[7] Pan S. Chen S. Huang Z. Zhou K. Zeng G. Tuerxun M. .Comparative analysis of clinical features of brucellosis in Kashi and Guangzhou: a retrospective multicentre study. BMC Infect Dis. (2025) (cited 2025 Sept 8);25. Available online at: https://pubmed.ncbi.nlm.nih.gov/40000956/10.1186/s12879-025-10479-4 PMC11863510 40000956

[8] Pan S. Wu Y. Zhou K. Gao J. Li J. Liu C. . A case of hepatic-splenic abscess in a non-endemic area of brucellosis: insights from complex infection with brucellosis. BMC Infect Dis. (2025) (cited 2025 Sept 8);25. Available online at: https://pubmed.ncbi.nlm.nih.gov/40481400/10.1186/s12879-025-11206-9 PMC12142888 40481400

[9] Ferreira AO Martins LN Marinho RT Velosa J. Spontaneous bacterial peritonitis by *Brucella* in a cirrhotic patient. BMJ Case Rep. (2013) (cited 2024 Aug 6);2013. Available online at: https://pubmed.ncbi.nlm.nih.gov/23563682/10.1136/bcr-2013-008629 PMC3645770 23563682

[10] Pan S Moming Z Awuti A Zhou K Tuerxun M Chong Y . Clinical insights into Brucella peritonitis: A comprehensive analysis of four cases. J Epidemiol Glob Health. (2024) 14:1300–4. doi: 10.1007/s44197-024-00287-5, PMID: 39186210 PMC11442879

[11] Z G Mem A Bh A Ha S Sb H. Risk factors, outcomes and time to detect positive blood culture among cases with acute brucellosis. Trans Royal Soc Trop Med Hyg (2022) [cited 2025 Nov 18];116. Available online at: https://pubmed.ncbi.nlm.nih.gov/34214996/10.1093/trstmh/trab093 34214996

[12] Yagupsky P Morata P Colmenero JD.Laboratory diagnosis of human brucellosis. Clin Microbiol Rev. (2019) (cited 2024 May 9);33. Available online at: https://pubmed.ncbi.nlm.nih.gov/31722888/10.1128/CMR.00073-19 PMC6860005 31722888

[13] Clinical and microbiological factors predicting outcomes of nonfermenting gram-negative bacilli peritonitis in peritoneal dialysis | Sci Rep. (cited 2025 Sept 8). Available online at: https://www.nature.com/articles/s41598-021-91410-0 10.1038/s41598-021-91410-0 PMC8192548 34112833

[14] Salzer WL. Peritoneal dialysis-related peritonitis: challenges and solutions. Int J Nephrol Renov Dis. (2018) 11:173–86. doi: 10.2147/IJNRD.S123618, PMID: 29928142 PMC6001843

[15] Ozisik L Akman B Huddam B Azap OK Bilgic A Sezer S . Isolated *Brucella* peritonitis in a CAPD patient Am J Kidney Dis. (2006). (cited 2025 Sept 8);47. Available online at: https://pubmed.ncbi.nlm.nih.gov/16632008/10.1053/j.ajkd.2006.01.028 16632008

[16] Zhu Y Shi L Zeng Y Piao D Xie Y Du J . Key immunity characteristics of diverse stages of brucellosis in rural population from Inner Mongolia, China. Infect Dis Poverty (2022) (cited 2025 Oct 24);11. Available onlin e at: https://pubmed.ncbi.nlm.nih.gov/35659087/10.1186/s40249-022-00989-7 PMC9167523 35659087

[17] Wang Y Yang S Han B Du X Sun H Du Y . Single-cell landscape revealed immune characteristics associated with disease phases in brucellosis patients. iMeta. (2024) 3:e226. doi: 10.1002/imt2.226 39135683 PMC11316929

[18] Roop RM Barton IS Hopersberger D Martin DW. Uncovering the hidden credentials of *Brucella* virulence. Microbiol Mol Biol Rev. (2021) 85:e00021. doi: 10.1128/MMBR.00021-19 33568459 PMC8549849

[19] Szeto CC Li PKT. Peritoneal Dialysis–associated peritonitis. Clini J Am Soc Nephrol. (2019) 14:1100–5. doi: 10.2215/CJN.14631218, PMID: 31068338 PMC6625612

[20] Editorial Board of the Chinese Journal of Infectious Diseases. Expert consensus on the diagnosis and treatment of brucellosis. Chinese Journal of Infectious Diseases. (2017) 35:705–10. doi: 10.5455/OVJ.2024.v14.i5.1

[21] Niu Q Zhao H Chen M Wu B Wang P Lu L . Brucella peritonitis in a patient on peritoneal Dialysis: Case Report and Literature Review. Perit Dial Int. (2018) 38:S64–8. doi: 10.3747/pdi.2018.00115 30315045

[22] Reis M Ribeiro C Gomes AM Santos C Lopes D Fernandes JC. Repeat and relapsing peritonitis microbiological trends and outcomes: A 21-year single-Center experience. Int J Nephrol. (2021) 2021:1–5. doi: 10.1155/2021/6662488, PMID: 33564478 PMC7867458

[23] Szeto CC Kwan BCH Chow KM Law MC Pang WF Leung CB . Repeat peritonitis in peritoneal dialysis: retrospective review of 181 consecutive cases. Clin J Am Soc Nephrol. (2011) 6:827–33. doi: 10.2215/CJN.05370610, PMID: 21183587 PMC3069376

